# Precise measurement of molecular phenotypes with barcode-based CRISPRi systems

**DOI:** 10.1186/s13059-025-03610-w

**Published:** 2025-05-25

**Authors:** Joseph H. Lobel, Nicholas T. Ingolia

**Affiliations:** 1 Department of Molecular and Cell Biology, University of California Berkeley, Berkeley, CA 94720 USA; 2 Center for Computational Biology and California Institute for Quantitative Biosciences, University of California, Berkeley, Berkeley, CA 94720 USA

**Keywords:** CRISPR/Cas9, Massively parallel reporter assay, CIBER-seq, Nonsense-mediated decay, Protein quality control

## Abstract

Genome-wide CRISPR-Cas9 screens have untangled regulatory networks driving diverse biological processes. Their success relies on interrogating specific molecular phenotypes and distinguishing key regulators from background effects. Here, we realize these goals by optimizing CRISPR interference with barcoded expression reporter sequencing (CiBER-seq) to dramatically improve the sensitivity and scope of genome-wide screens. We systematically address technical factors that distort phenotypic measurements by normalizing expression reporters against closely matched promoters. We use our improved CiBER-seq to accurately capture known components of well-studied RNA and protein quality control systems. These results demonstrate the precision and versatility of CiBER-seq for dissecting cellular pathways.

**Supplementary Information:**

The online version contains supplementary material available at 10.1186/s13059-025-03610-w.

## Background

Comprehensive, genome-scale reverse genetic screens have successfully uncovered the molecular underpinnings of diverse biological processes [1]. CRISPR-Cas9 is a versatile tool for reverse genetics that can be targeted by guide RNAs (gRNAs) to create programable genome-wide knockdowns or knockouts [2–4]. By determining which guides alter a specific cellular behavior, one can identify the responsible regulatory genes and prioritize these for mechanistic studies. These approaches have been widely used, and increasing the sensitivity and range of phenotypes accessible to these platforms would have broad benefits [1].

A central challenge in CRISPR-based reverse genetics is coupling genetic perturbations to their biological effects on a genome-wide scale. Towards this end, our lab combined *C*RISPR interference (CRISPRi) with *B*arcoded *E*xpression *R*eporter *seq*uencing (CiBER-seq) to directly and quantitatively measure diverse molecular phenotypes by high-throughput sequencing [5]. CiBER-seq uses a library of gRNAs that are linked with a transcribed reporter containing a unique, guide-specific barcode; guides that change expression of the reporter—for example, by perturbing a regulator—will change expression of their individual, guide-specific barcode. Measuring reporter expression changes by deep sequencing of the barcodes allows for simple, pooled, genome-wide interrogation of numerous biological processes [5–7]. Reporter expression can assess many nuanced phenotypes that are inaccessible by simply measuring cell growth and survival. Furthermore, quantifying reporter expression through deep sequencing offers substantial advantages compared to fluorescent protein reporters analyzed by flow sorting and sequencing, which discretizes effects into specific bins and has limited throughput. More generally, RNA reporters are better equipped to directly address the regulation of RNA metabolism and post-transcriptional processes [5, 7–10]. Thus, coupling CRISPRi with barcode measurements enables quantitative reverse genetic screens across a broader range of phenotypes and cellular conditions.

While CiBER-seq and other barcode-based CRISPRi systems have emerged as powerful tools to assess genotype-to-phenotype relationships, these approaches are still in their infancy [5, 7, 8]. Like most screening technologies, CiBER-seq can suffer from background that arises when genetic perturbations affect the reporter system itself rather than the biological process under investigation, leading to the spurious appearance of phenotypic effects that obscures interesting candidates. Initial versions of CiBER-seq drew inspiration from massively parallel reporter assays (MPRAs) that infer variant effects by normalizing RNA barcode expression to DNA barcode levels [11–13]. This normalization strategy works well in MPRAs, where each variant experiences a similar cellular environment and so the reporter system is consistent between different library members. However, CiBER-seq introduces distinct genetic perturbations to each cell, and cells expressing guides that affect bulk transcription can have altered RNA-to-DNA ratios independently of the queried phenotype. Accordingly, previous iterations of CiBER-seq found many significant guides that affected core mRNA transcriptional machinery and likely reflect a general impediment to any reporter expression rather than relevant regulation [5, 7]. Technical factors can also add noise or otherwise distort phenotypic measurements. In sequencing-based genetic screens, RNA and DNA barcode abundance measurements require different isolation and library preparation that can lead to technical variation between the two samples that alters the RNA-to-DNA ratio and thus the inferred phenotype [14]. Overcoming these technical and systems-level effects would enhance the ability to quantitatively define regulators of cellular responses.

Here, we dramatically improve the sensitivity of barcode expression reporters and expand their scope to interrogate diverse molecular processes. We essentially eliminate background in CiBER-seq experiments by expressing RNA barcodes from two closely matched promoters and further reduce noise through precise, single-copy integration of these reporters. This generalizable approach enables accurate dissection of genetic networks controlling diverse protein and RNA-level phenotypes. We use this system first to profile genetic regulators of a potent protein degradation signal, finding the established ubiquitin–proteasome system known to drive its turnover. We then adapt CiBER-seq to directly examine mRNA quality control and identify the conserved nonsense-mediated decay factors that are required to degrade a transcript containing a premature termination codon. In both cases, our optimized platform finds known regulators with exquisite precision and minimal background. These simple and effective improvements for barcode-based CRISPRi screens are readily adaptable to investigate genetic regulators across a range of molecular phenotypes.

## Results

### RNA barcodes expressed from matched promoters eliminates background in barcode-based CRISPRi screens

**Fig. 1:**
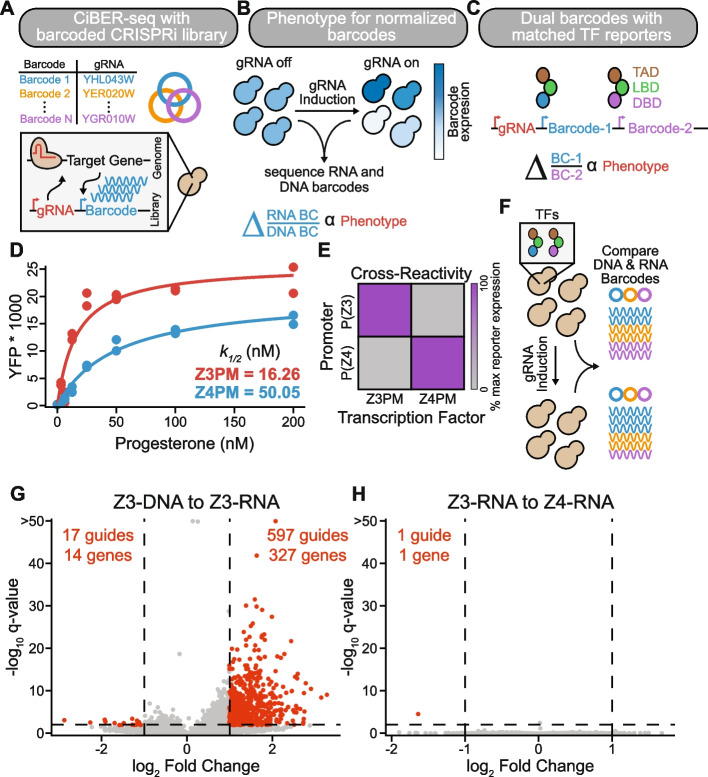
Eliminating background in barcode-based genetic screens. **A** Schematic of paired guide-barcode libraries for CRISPRi screening. **B** Workflow for CiBER-seq screen and determination of phenotypic effects. Sequencing libraries are prepared from samples before and after guide induction, normalizing RNA barcodes to DNA barcodes. **C** An optimized CiBER-seq platform using closely matched transcription factors that each express a barcode from similar promoters with common genetic dependencies. TAD: Transcriptional activation domain, LBD: Ligand-binding domain, DBD: DNA-binding domain. **D** Characterization of the dose response of each hormone-inducible transcription factor by flow cytometry of yeast transformed with a YFP expressed from the cognate promoter. Mean YFP was fit to a simple binding isotherm for biological replicates, see the “Methods” section (*n* = 2). **E** Cross reactivity of Z3PM or Z4PM expressing YFP from a P(Z3) or P(Z4) promoter. YFP expression is normalized to the transcription factor and its cognate promoter (*n* = 2). **F** Schematic for evaluating technical variations between DNA and RNA barcode-based comparisons. All barcodes are isolated from the same sample before and after guide induction. **G** Analysis of genome-wide CiBER-seq screen with Z3PM RNA barcodes normalized to DNA barcodes levels. Each point is a single guide, with significant guides colored red. A *q*-value < 0.01 and > 1 log_2_ fold change and is represented by dashed lines. **H** Same screen as in **G**, except Z3PM barcode expression was normalized to the control barcodes driven by Z4PM

CiBER-seq is a massively parallel reporter approach that measures how CRISPRi-based gene knockdown affects a transcriptional reporter through deep sequencing of an expressed mRNA barcode [5]. These barcodes are embedded within the 3′ UTR of a reporter transcript and uniquely linked to a guide on the same plasmid (Fig. 1A) [5]. Comparing the abundance of each expressed RNA barcode before and after induction of guides reveals how the CRISPRi knockdown of a gene influences expression of its associated reporter (Fig. 1B). Accurate expression measurements require normalization of barcode RNA abundance to correct for the abundance of cells containing that barcode within the population, which varies between experiments and can change due to growth defects. Typically, DNA barcodes—which should be present at one copy per cell—are sequenced to control for these variations (Fig. 1B). RNA-to-DNA ratios have been used in previous CiBER-seq screens by our lab and in other barcode-based CRISPRi screens to interrogate diverse biological regulatory programs [5, 7, 8]. While these methodologies have yielded valuable discoveries, improving the precision and scope of these technologies promises to more accurately decipher genetic networks underlying complex cellular processes.

A key challenge in any high-throughput screen is distinguishing the biological phenotype of interest from effects caused by technical aspects of the system. In massively parallel reporter assays, differences in sample preparation between RNA and DNA libraries can distort expression estimates. Additionally, in CiBER-seq, guides that affect core cellular processes may influence barcode DNA abundance or RNA expression, leading to biologically irrelevant changes in the measured RNA-to-DNA ratio. Indeed, initial CiBER-seq screens from our lab using RNA-to-DNA ratios found numerous guides that changed this ratio significantly, but seemed unlikely to affect the examined phenotypes [5]. These experiments, which probed the integrated stress response, identified known components of this well-established pathway, albeit with high background from guides that could instead affect the reporter system itself. For example, perturbations to general transcription machinery produced significant effects, likely by influencing barcode expression itself rather than the queried phenotype (Additional file 1: Fig. S1 A–B) [5]. While guides affecting general transcription can easily be distinguished as technical background from a screen with an RNA expression readout, many others were ambiguous. Collectively, these technical differences between RNA and DNA barcodes can cause high background that masks sought-after targets and necessitates laborious curation and validation of individual candidates. To mitigate these effects, previous work from our lab expressed reporter and normalizer barcodes from two different promoters. These RNA-to-RNA comparisons controlled for many irrelevant and systemic guide effects and eliminated technical differences that arise from separately handling plasmid DNA and mRNA. Nonetheless, we found significant effects from hundreds of guides that targeted general transcription machinery, which we attributed to differences in the genetic requirements of the reporter and normalizer promoters rather than a specific connection to the phenotype of interest [5, 7]. Correcting for these systems-level effects would increase the sensitivity and robustness of CiBER-seq.

We reasoned that barcodes expressed from closely matched promoters would share the same genetic dependencies and measuring the ratio between these barcodes should eliminate much of the systematic and technical variation in CiBER-seq (Fig. 1C). In such a system, knockdown of genes that affect technical aspects of barcode expression should be experienced equally by the reporter and normalizer and produce no change in barcode ratio. Achieving this idealized setup requires promoters that respond to biologically interesting regulation while otherwise sharing similar genetic requirements. We turned to established synthetic transcriptional reporters driven by chimeric transcription factors that exclusively bind their cognate synthetic promoter [15]. We used two different promoters derived from the same core *GAL1* sequence that differ only in the binding sites for distinct, heterologous zinc-finger DNA binding domains, termed “Z3” and “Z4”. These DNA-binding domains are incorporated into artificial hormone inducible transcription factors, Z3PM and Z4PM, that share a common progesterone binding and Msn2 *trans*-activation domains (Fig. 1C) [15, 16]. We also appended a PEST degron domain to increase transcription factor turnover and ensure protein levels more accurately track the short half-lives of mRNAs in yeast [17, 18]. We confirmed that these transcription factors had similar hormone responses, are highly specific for their cognate promoter, and show no growth defects when induced, making them ideal candidates to drive barcode expression in CiBER-seq (Fig. 1D–E, Additional file 1: Fig. S1 C–F).

We assessed how comparing barcodes expressed from these closely matched reporters could minimize background effects seen in previous iterations of CiBER-seq (Additional file 1: Fig. S1 A-B) [5]. We performed a screen using reporter and normalizer barcodes driven by the Z3PM or Z4PM transcription factors, respectively, with no additional differences between them, so that any apparent phenotypic effects could be attributed unambiguously to systemic background. To directly contrast conventional RNA-to-DNA measurements with our optimized CiBER-seq, we carried out deep sequencing of both DNA and RNA barcodes from the same samples (Fig. 1F). We transformed yeast expressing both Z3PM and Z4PM with a plasmid-based, genome-scale guide library containing barcoded reporters driven by these transcription factors. We grew two biological duplicate cultures at a constant density, collected cells before and after ~ 9 h of guide induction (~ 6 doublings), isolated RNA and DNA in parallel, and counted barcodes by deep sequencing (Additional file 1: Fig. S1G) [19]. We obtained > 14 million reads for each sample, and all read counts were within ~ twofold of each other, providing similar sequencing depth and statistical power for all comparisons. We then identified guides that caused significant changes in reporter expression using established linear models for massively parallel reporter assays (with the *mpra* package), which determines significance by assessing how barcode ratios change after guide induction while controlling for technical variability in counts [20].

Knockdown of hundreds of genes changed the RNA-to-DNA barcode ratio. We saw many significant effects from guides involved in DNA replication or RNA metabolism, similar to our results in prior CiBER-seq screens that compared RNA and DNA barcodes (Fig. 1G, Additional file 1: Fig. S1 A–B and Fig. S2) [5]. In stark contrast, comparing Z3- and Z4-driven RNA barcodes revealed only one significantly different guide (Fig. 1H). Since both comparisons were made using the exact same biological samples, we conclude that the high background observed in RNA-to-DNA measurements results from technical effects, which can distort signal and obscure discovery of regulators driving the phenotype of interest [5, 7]. We also confirm that effects observed from these guides in previous CiBER-seq experiments reflect false positives, as we had hypothesized (Additional file 1: Fig. S1 A–B) [5, 7]. Closely matched promoters provide a simple solution to dramatically reduce background in CiBER-seq and similar approaches, and should thereby enable more precise identification of relevant targets in reverse genetic screens.

### High-efficiency genomic integration of CiBER-seq libraries

Like many other massively parallel experiments conducted in yeast, CiBER-seq relies on libraries expressed from autonomously replicating plasmids. Plasmid loss and copy number variation, arising due to imperfect plasmid segregation, introduces technical noise into reporter measurements [21, 22]. Furthermore, continuous selection is required to maintain plasmids, which constrains the growth conditions that can be used. While genomic integration solves these limitations, homology-directed integration into the yeast genome occurs at much lower efficiency than plasmid transformation; this bottleneck limits its application in massively parallel experiments where large numbers of transformants are needed to span diverse libraries. Double-stranded breaks created by CRISPR-Cas9 or other nucleases can enhance integration, but this process is not benign, and using CRISPR-Cas9 for guide library integration conflicts with using Cas9 derivatives to create genetic perturbations [22, 23]. Site-specific recombinases enable genomic integration into a pre-established genomic landing pad without double-stranded breaks, and the tyrosine recombinase Cre has been used to integrate complex libraries of up to ~ 500,000 lineages in budding yeast, but remains too limited to cover libraries with > 100,000 unique members [24]. Serine recombinases have emerged as attractive alternatives for genomic integration, and in human cells, Bxb1 and other members of this family have been utilized to create genomic landing pads that support high-efficiency integration of complex libraries [25–27]. Bxb1 works efficiently in yeast, and we reasoned that a similar landing pad might allow high-diversity, chromosomally integrated libraries in CiBER-seq screens [28].

**Fig. 2:**
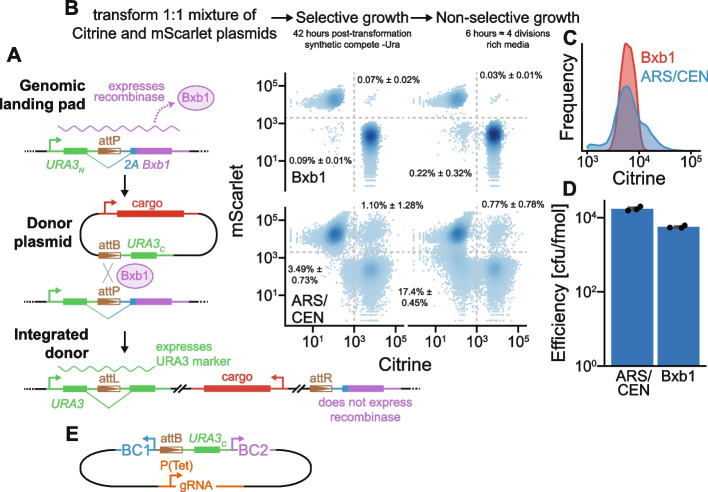
High-efficiency, single-copy integration of reporters with a Bxb1 recombinase-based system. **A** Schematic of yeast-based Bxb1 integration system. Bxb1 is constitutively expressed until recombined with a donor plasmid, which reconstitutes a *URA3* selectable marker. **B** Representative flow cytometry of yeast transformed with a mixture of plasmids encoding yECitrine or yEmScarlet-I through plasmid-based or Bxb1-mediated recombination approaches. Fluorescence was recorded both before and after removal of selective pressure. Percentage of cells in the double-positive and double-negative quadrants are shown (mean ± standard deviation, *n* = 3). **C** Distribution of yECitrine fluorescence of yeast transform through different approaches. **D** Transformation efficiency of plasmid-based or Bxb1-mediated recombination approaches (*n* = 3). **E** Diagram of CiBER-seq library with Bxb1 integration system

We created CiBER-seq libraries of donor plasmids with guide RNAs and barcoded reporters that could be integrated into a single genomic landing pad (Fig. 2A). We drew from other systems to provide streamlined integration and stringent selection of transformants. Specifically, our landing pad constitutively expresses a yeast-optimized Bxb1 recombinase gene that is inactivated by integration of a donor plasmid, eliminating the need for additional components or recombinase induction. Integration of the donor plasmid reconstitutes a *URA3* selection marker gene from two halves that are split between the landing pad and the donor, ensuring marker expression only when the donor is integrated specifically at the landing pad [24, 29]. We see no background growth in selective conditions in the absence of on-target integration, consistent with previous studies using similar split markers.

We then tested whether landing pad integration resolved the sources of noise seen in autonomous plasmids. We transformed yeast with an equal mixture of plasmids expressing the yellow fluorescent protein yECitrine and the red fluorescent protein yEmScarlet-I to compare autonomous plasmids with Bxb1 donors (Fig. 2B). We carried out bulk selection in liquid culture and then analyzed the transformed populations by flow cytometry. After selection, virtually all yeast transformed with the Bxb1 donor exclusively expressed one fluorescent protein, which persisted for several generations after selection was removed. In contrast, plasmid transformation yielded a large population of cells that lacked plasmid and quickly expanded when selection was relaxed. Furthermore, a substantial number of yeast expressed both fluorescent proteins simultaneously, indicating the presence of multiple, distinct plasmids within the cell. In the context of a complex library transformation, such as a CiBER-seq experiment, yeast containing two different library plasmids would contribute to technical noise. This difference in plasmid levels also produced more variation in fluorescence protein expression than Bxb1-mediated integration (Fig. 2C). Importantly, Bxb1-mediated integration was efficient enough for large-scale transformation of complex libraries and was within ~ threefold of autonomous plasmids transformation (Fig. 2D). Because recombinase-mediated integration at a genomic landing pad maintains high transformation efficiency while ameliorating several technical challenges posed by plasmid libraries, we constructed a barcoded guide library compatible with Bxb1 integration for subsequent genome-wide CiBER-seq experiments (Fig. 2E).

### Optimized CiBER-seq identifies regulators of a degron

**Fig. 3:**
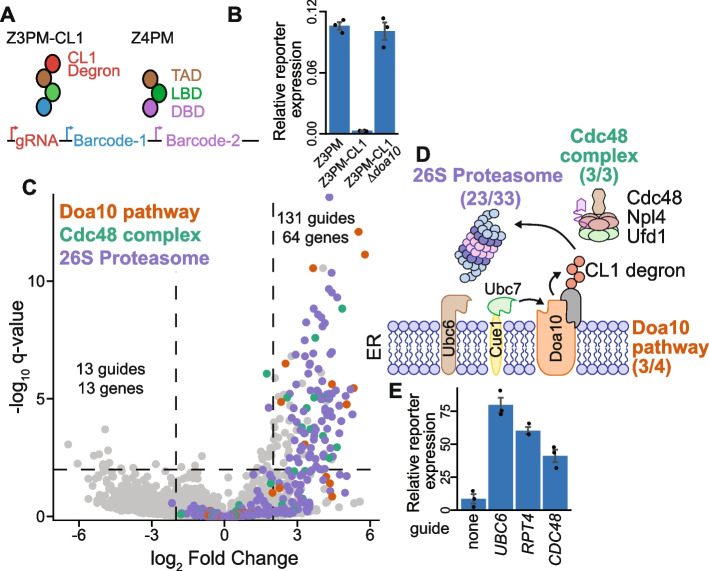
Precise identification of degron regulators with optimized CiBER-seq. **A** Schematic for CiBER-seq to characterize regulators of the CL1 degron. **B** RT-qPCR of reporter transcripts expressed by Z3PM or Z3PM-CL1 in a wildtype or *∆doa10* background, compared to the normalizer transcript (*n* = 3). **C** Analysis of genome-wide CiBER-seq screen for regulators of the CL1 degron. Each point is a single guide and colored based on molecular function in the legend. Significant and robust guides were assessed by a *q*-value < 0.01 and > 2 log_2_ fold change, which is represented by dashed lines. **D** Schematic of the established CL1 turnover pathway with yeast names displayed, with the number of guides targeting proteins in each pathway or complex displayed. **E** RT-qPCR of reporter barcodes expressed by Z3PM-CL1 with guides induced (*n* = 3)

We aimed to benchmark this optimized CiBER-seq platform and demonstrate its ability to characterize diverse molecular phenotypes with high precision by surveying genetic modifiers of targeted protein degradation. The well-studied CL1 degron is ubiquitinated by the E3 ligase Doa10 and subsequently delivered by Cdc48 to the 26S proteasome for degradation; we expected that depleting components of these protein complexes would stabilize the CL1 degron [30, 31]. We appended the CL1 degron to the Z3PM transcription factor to destabilize it and thereby decrease Z3-driven barcode expression (Fig. 3A). Indeed, Z3-driven barcode expression was ~ 40x lower than expression of barcodes driven by the untagged Z4PM construct. Reduced Z3PM barcode expression was fully rescued when Doa10 was knocked out, confirming that our reporter detects genetic changes in CL1 degradation and thus could be used for CiBER-seq profiling of post-translational regulation (Fig. 3B).

We then performed a genome-wide CiBER-seq screen to characterize factors controlling the stability of the CL1 degron. We integrated a dual-reporter, barcoded guide library using Bxb1-mediated recombination into yeast expressing both Z3PM-CL1 and Z4PM along with CRISPRi machinery, sequenced RNA barcodes, and quantified changes in barcode ratios after guide induction (Additional file 1: Fig. S3 A). As expected, many guides that stabilized the reporter transcription factor targeted genes were previously found to be important for turnover of the CL1 degron through knockout or temperature-sensitive alleles (Fig. 3C-D). For example, we identified three out of the four components of the Doa10 E3 ligase system, including the sequential E2 enzymes Ubc6 and Ubc7 [32]. We also identified guides targeting 22/33 components of the 26S proteasome and each member of the Cdc48 complex (Fig. 3D). Several additional components of these complexes were targeted by guides that fell just outside our significance cutoff. Overall, this approach identified 131 guides targeting 64 genes that stabilized the reporter transcription factor, ~ 78% of which were involved in either the 26S proteasome, Doa10 ubiquitination, or Cdc48 complex, demonstrating the high precision of our optimized CiBER-seq approach [30]. In contrast, guides that appeared to further destabilize the Z3PM-CL1 degron likely represent a low level of noise or background, as individually testing three such guides did not decrease reporter expression (Additional file 1: Fig. S3B). These guides only minimally crossed our statistical cutoff, and no gene was targeted by more than one such guide, whereas we saw on average more than two guides per gene for CL1 stabilization (Fig. 3D). This specific recovery of known CL1 degradation factors contrasts sharply with the high background in previous RNA-to-DNA based CiBER-seq experiments (Additional file 1: Fig. S1 A–B) [5].

A major advantage of CRISPRi in genome-scale screening is its ability to target essential genes and produce non-lethal loss-of-function phenotypes that reveal essential gene function. For example, we found numerous guides that stabilized CL1 by targeting many components of the essential 26S proteasome or Cdc48 complex. We chose several of these guides, including those targeting both essential genes and non-essential components of the degradation pathway, and tested their effects individually. In each case, knockdown of these genes increased reporter levels controlled by the Z3PM-CL1 construct, consistent with stabilization of the degron (Fig. 3E). Furthermore, CiBER-seq identified genes not previously known to influence CL1 turnover, and knockdown of these genes likewise stabilized reporter expression (Additional file 1: Fig. S3B). Comprehensive recovery of the known CL1 degradation pathway, and nomination of new factors, demonstrates the exquisite specificity and sensitivity of our optimized CiBER-seq platform for mapping the genetic drivers of regulated protein degradation.

### Expanding optimized CiBER-seq to characterize regulators of RNA phenotypes

Given our success in identifying the full pathway responsible for CL1 degradation, we next wanted to further expand the phenotypes we could address with CiBER-seq. We reasoned that our approach could examine post-transcriptional regulatory processes, which shape gene expression by modulating mRNA half-lives or degrading faulty messages. Despite their central role in cellular processes, RNA-level phenotypes are usually measured indirectly. Typical studies couple problematic mRNAs to expression of a fluorescent protein, which is used as a proxy for transcript abundance [33–39]. While valuable, these systems do not distinguish the separate contributions of translational control and transcript stability to the observed response. Our understanding of these complex post-transcriptional regulatory events would be aided by methods that more directly interrogate regulators of RNA-level processes.

**Fig. 4:**
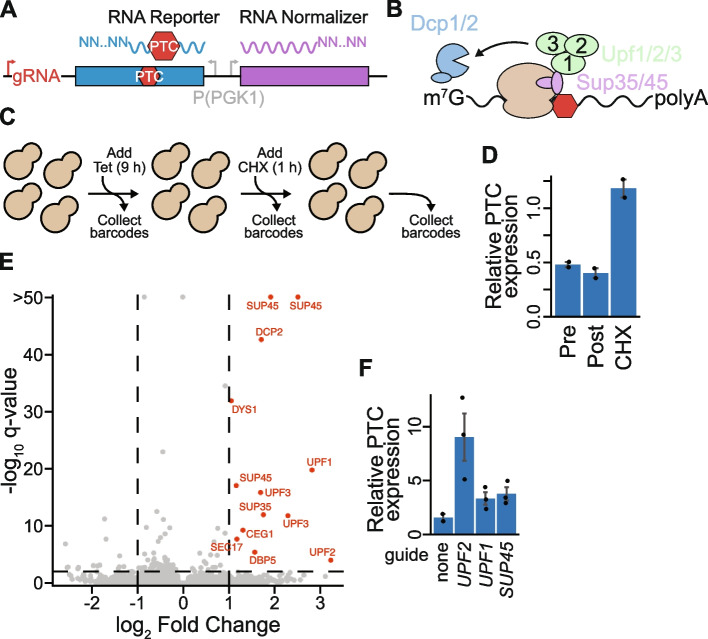
CiBER-seq directly measures regulators of an RNA quality control pathway. **A** Schematic for using CiBER-seq to interrogate RNA-level phenotypes. Barcodes are embedded in the 3′ UTR of a reporter and normalizer, which are constitutively expressed from identical promoters. **B** Proteins involved in NMD, with yeast gene names displayed. **C** Workflow for CiBER-seq to investigate regulators of NMD and their dependency on active translation. **D** RT-qPCR of the NMD reporter, compared to the normalizer transcript without the PTC, at all steps during CiBER-seq (*n* = 2). **E** Analysis of genome-wide CiBER-seq screen for regulators of NMD. Guides with a *q*-value < 0.01 and > + 1 log_2_ fold change are labelled in red, with thresholds represented by dashed lines. **F** RT-qPCR of PTC-containing mRNA compared to normalizer with guides induced (*n* = 2–3)

CiBER-seq is well positioned to break this barrier because it infers guide effects by measuring RNA barcodes. Inserting barcodes within the 3′ UTR of a problematic mRNA would allow a genome-wide CRISPRi screen that directly assesses how each guide influences levels of the reporter transcript (Fig. 4A). As a test case, we chose to characterize regulators of nonsense mediated decay (NMD), a translation-coupled process that triggers destruction of messages harboring a premature termination codon (PTC). During NMD, release factors recognize the PTC within the ribosome and assemble Upf proteins, which recruit the decapping machinery to trigger degradation of the message (Fig. 4B) [40]. To profile regulatory factors that control turnover of a NMD substrate, we designed a reporter mRNA that possessed a PTC and a normalizer that lacked one. We constructed a CiBER-seq library that embedded the barcodes within the 3′ UTRs of the NMD and control reporters, where they serve as a direct measurement of mRNA levels. As before, we sought to minimize background by ensuring both transcripts were expressed from matched promoters (Fig. 4A). We then performed a genome-wide CiBER-seq screen by sequencing barcodes from samples taken before and after guide induction. Unlike protein-based reporters, direct measurements of RNA allowed us to additionally interrogate phenotypic changes that occur when translation is arrested. Because a hallmark of NMD is its dependence on active translation, we assessed this requirement by also collecting barcodes 1 h after treating cells with cycloheximide, which stabilized the PTC containing reporter (Fig. 4C–D and Additional file 1: Fig. S4 A) [41].

Again, our optimized CiBER-seq screen precisely identified key NMD genes with minimal background. We found strong NMD suppression from guides targeting both Sup35 and Sup45 (which encode the release factors eRF1 and eRF3), the three Upf proteins, and the decapping protein Dcp2 (Fig. 4B–E). More broadly, these guides were strongly enriched for NMD and translation termination gene ontology terms (Additional file 1: Fig. S4B). Indeed, 10 out of the 13 guides that stabilized reporter expression targeted genes with known roles in NMD, while guides that appeared to further destabilize the PTC reporter did not share any common genes and lacked any significant gene ontology terms. Known NMD factors lost activity when translation was arrested with cycloheximide, consistent with the requirement for active translation to degrade PTC-containing messages (Additional file 1: Fig. S4 C–D) [40]. This work highlights how CiBER-seq can uncover molecular phenotypes in the absence of ongoing translation, something not possible with fluorescent protein-based or survival screens. Experiments performed with individual guides confirmed that these knockdowns stabilized the PTC reporter and recapitulated the rank order of phenotypes observed in our CiBER-seq screen, while guides that further destabilized the reporter showed minimal effects (Fig. 4F and Additional file 1: Fig. S4E). Curiously, our screen did not find any guides targeting Dcp1*,* an obligate partner of Dcp2. We tested individual guides targeting the *DCP1* locus and found that they did not reduce expression of its mRNA, demonstrating how false negatives within CiBER-seq screens can arise from poor knockdown efficiency of guides (Additional file 1: Fig. S4E–F). We again observed minimal background effects in profiling this distinct phenotype, revealing the general improvement provided by matched promoters relative to previous normalization approaches for eliminating background in barcode-based CRISPRi platforms (Fig. 4E and Additional file 1: Fig. S1 A–B). Combining our optimized CiBER-seq with direct measurements of RNA reporters enables CRISPRi approaches to quantitatively and precisely interrogate both RNA and protein-level molecular phenotypes.

## Discussion

The combination of CRISPRi genetic perturbations and barcoded expression reporters holds clear promise as a widely applicable tool for biological discovery. Increasing the range of processes that can be assessed with barcoded reporters and reducing noise and background in these measurements would further enhance these powerful techniques. Here, we realize the full potential of CiBER-seq platforms by addressing the major sources of background with simple and generalizable modifications. We find that using RNA reporter and normalizer barcodes, driven from closely matched promoters, eliminates irrelevant technical effects across multiple screens. Recombinase-mediated integration further ensures stringent isolation of single guides in each cell. We combine these elements in CiBER-seq screens that accurately and comprehensively identify known regulators in post-transcriptional and post-translational control pathways. These results illustrate how this optimized CiBER-seq strategy provides a broadly applicable platform for genome-wide screens.

While genome-wide knockout or knockdown approaches have been invaluable for mapping regulatory networks, background effects can obscure true biological signals in these high-throughput experiments [42]. These artifacts can arise from both technical and biological differences between how reporter and normalizers are treated throughout the experiment. For example, previous iterations of CiBER-seq profiled established biological responses using RNA to DNA barcode normalization and found dozens of guides that changed reporter expression but lacked an obvious connection to the queried phenotype. Even experiments that exclusively used RNA barcodes found numerous guides whose apparent phenotype likely reflected the differing genetic requirements of the distinct promoters driving expression of each barcode [5, 7]. We find that expressing RNA barcodes from closely matched promoters eliminates such background both by ensuring the reporter and normalizer respond similarly to perturbations that affect their shared genetic dependencies and reducing technical variation in sample processing. Supporting this notion, we infer dramatically different guide activities within the same sample depending on the normalization approach. Even in experiments using highly similar transcription factors, hundreds of guides appeared active when normalizing RNA-to-DNA barcodes. In contrast, RNA-to-RNA barcode comparisons show no significant effects and argues that this design minimizes systems-level effects in barcode-based CRISPRi screens. We then extended this concept to accurately identify known genes across two distinct phenotypes. This facile improvement in reporter design promises to improve identification of regulators through genome-wide CRISPRi knockdown screens.

The improved quantitation and precision of this optimized CiBER-seq approach addresses a major challenge in functional genomics. In profiling regulation of the CL1 degron, we found many genes with established roles in this pathway with minimal background. Our approach also offers to expand our ability to interrogate regulators of critical biological processes by directly interrogating post-transcriptional regulation of RNA reporters. Using NMD as a model, CiBER-seq highlighted the known NMD factors and confirmed their dependence on translation. This platform now opens opportunities to directly assess RNA-level phenotypes with CRISPR screens and paves the way to examine post-transcriptional processes that do not require translation, such as nuclear RNA decay or noncoding RNA metabolism [43–46]. In both cases, these barcoded reporter screens complement FACS-based methods, such as Sort-seq analysis of fluorescent protein reporters or HCR-flowFISH profiling of endogenous mRNAs, for dissecting regulatory networks [9, 47]. Sequencing-based readouts, however, offer several distinctive advantages because they require only bulk RNA samples for each experimental conditions and capture a broad range of activity rather than discretizing signal into bins [9, 47]. Our improved CiBER-seq system uses a sequencing readout to provide pooled functional genomic profiling of diverse molecular phenotypes.

While this work provides a general blueprint for applying CiBER-seq, additional considerations may arise in specific applications. Closely matched sample processing and reporters with similar genetic dependencies reduce noise, and these should be carefully evaluated when adapting this technique to other phenotypes and organisms. First, artificial “orthogonal” transcription factors have been developed for numerous model systems, and these can be fused to a query protein of interest in order to interrogate its regulators [48]. By integrating such factors into endogenous loci by homology-directed repair, our matched-reporter strategy can be applied to study diverse biological systems while minimizing background signal [7]. Investigators should confirm proteins retain function when fused to a reporter transcription factor to ensure the tagged protein retains biological activity. Second, our screens used RNA or protein appendages that dramatically reduce reporter expression and were correspondingly adept at detecting perturbations that increase barcode counts. We expect screens that examine stabilizing elements will find knockdowns that reduce barcode expression by disrupting their stabilizing effects. Third, many of the concepts outlined here are directly transferable to screens in higher eukaryotes, such as mammals, and will help minimize background in pooled sequencing-based CRISPRi experiments [49]. Incorporating additional reporters or perturbations can further expand the range of processes accessible to barcode-based genetic screens. Last, because CiBER-seq is a sequencing-based, massively parallel reporter assay across genetic perturbations, we anticipate that it can be combined with single-cell sequencing to interrogate specific molecular processes in complex cellular environments [50]. These platforms will enable precise dissection of the intricate genetic networks controlling cellular behaviors from the biochemical to organismal scale.

## Conclusions

Our improvements to CiBER-seq provide a generalizable approach that robustly distinguishes relevant guides from technical background in barcode-based genome-wide CRISPR/Cas9 screens. The versatility of barcode-based reporters allows profiling of both protein and RNA-level phenotypes with high sensitivity. These simple improvements can be easily adapted to interrogate the genetic requirements underlying a broad range of cellular responses.

## Methods

### Yeast strain construction

All *S. cerevisae* strains used were derived from BY4742, grown at 30 °C, and transformed with the LiAc/PEG method as described previously [51]. Unless otherwise stated, all yeast were collected by centrifugation at 3000 g for 5 min at room temperature. Codon optimization of Bxb1 was performed using iχnos [52]. Successful integration of reporter transcription factors, Cas9/TetR, or the attP landing pad was verified by PCR amplification across sequence junctions. For constructing PEST-Z3PM and PEST-Z4PM containing strains, these transcription factors were cloned into separate easyclone 2.0 vectors through Gibson assembly to make pJL200 and pJL201 [53]. The CL1 degron was appended to the C-terminus of PEST-Z3PM to make pJL259. In all cases, vectors were linearized through PluTI (NEB, R0713S) digestion for 1 h at 37 °C, transformed into log phase yeast using the LiAc/PEG approach, and selected for on the appropriate media. A complete list of strains, sequencing primers, and plasmids used in this study are available in Additional file 2: Table S1.

### Transcription factor hormone response

Yeast expressing transcription factors and promoters were grown in SCD-Ura supplemented with the appropriate amount of progesterone (Sigma-Aldrich, P0130). Overnight cultures of yeast were backdiluted to OD_600_ = 0.1 and grown until OD_600_ = 0.5 before being collected by centrifugation, washed once with PBS, and then fixed in 4% paraformaldehyde/PBS solution at room temperature in the dark for 15–60 min. Fixed cells were pelleted, resuspend in PBS, and stored at 4 °C until ready for use. For testing transcription factor orthogonality, strains with genomically integrated Z3PM or Z4PM and plasmid containing P(Z3)-YFP or P(Z4)-YFP were grown in SCD-Ura with 200 nM progesterone as described above. Flow cytometry was performed on a BD LSRFortessa cell analyzer using the appropriate cytometer settings, gated for single cells, and analyzed using *flowCore* in R [54]. Hormone response curves and k_1/2_ were fit to the following equation in R:

$${YFP}= \frac{{A}_{\text{max}}*\left[H\right]}{{k}_{1/2}+[H]}$$

where *H* is the progesterone concentration and *A*_max_ represents a fit variable to the maximum YFP. All measurements were derived from 2 to 3 biological replicates.

### Optimized CiBER-seq reporter design and library construction

We chose to use matching P(Z3) and P(Z4) promoters that are recognized by the Z3- and Z4-DNA binding domains, respectively [15]. For protein-level phenotypes, these promoters drove expression of reporter mRNAs that encoded yeast optimized YFP and CFP [55]. The CFP transcript contained several synonymous mutations to allow specific primer binding during library amplification. For the NMD reporter, divergent *PGK1* promoters drove expression of a reporter mRNA encoding a YFP with a premature termination codon or a full-length yeast optimized mScarlet reporter [56]. In both cases, reporters were flanked by homology to the dual barcoded-guide library and BciVI sites that allowed excision of the reporters for library construction.

All CiBER-seq screening libraries were derived from a previously reporter library containing an sgRNA paired with two unique 25 nucleotide barcodes [5]. A step-by-step description of guide cloning, barcode insertion, and barcode-guide pairing are detailed elsewhere [57]. For cloning reporters into the barcoded libraries, 2 µg of the sgRNA-barcode library was linearized with AscI (NEB, R0558S) and, separately, 2 µg of the plasmids containing the reporters and promoters (pJL206 or pJL261) were linearized with BciVI (NEB, R0596S) for 1 h at 37 °C. The digestions were purified by spin column (Qiagen, 28104) according to the manufacturer’s instructions. 480 ng of the sgRNA-barcode library and 230 ng of the reporter construct were mixed in separate 20 µL NEBuilder HiFi assembly reactions (NEB, E2621S) for 1 h at 50 °C. The assembly was then purified with the Monarch PCR & DNA cleanup kit (NEB, T1030S), eluted in 6 µL of water, and electroporated into 5 × 20 µL MegaX DH10B T1^R^ Electrocomp cells (ThermoFisher, C640003) according to the manufacturer’s protocol. Cells were shaken at 37 °C for 1 h in recovery media, transferred into 200 mL LB + Kan + Cam, and grown overnight at 28 °C to OD_600_ = 3.0. Library coverage was estimated by serial dilution of transformations before overnight growth onto selective plates to ensure > 100 unique transformants for each barcode in the library. CiBER-seq libraries were then purified from overnight cultures by Midiprep (Qiagen 12143) according to the manufacturer’s protocol.

### CiBER-seq screens

The reporter strains were transformed with the appropriate library by inoculating 250 mL YEPD with yeast to OD_600_ = 0.1 and growing cells for 2–3 doublings. Cells were collected by centrifugation at 3000 g for 10 min at room temperature and transformed using a 30 × scaled up LiAc/PEG transformation protocol with > 40 µg of the appropriate CiBER-seq library for each of two biological replicates [51]. After heat shock at 42 °C for 45 min, cells were collected by centrifugation at 3000 g for 5 min, transferred to 500 mL SCD-Ura, and grown at 22 °C for ~ 2 days until OD_600_ = ~ 2. After reaching the target density, 210 OD_600_ units were harvested by centrifugation and stored at − 80 °C in SCD-Ura + 15% glycerol until ready for use. We estimated the number of unique transformants by serial dilution and plating on SCD-Ura, ensuring > 10 × coverage of each barcode in the library.

To perform the CiBER-seq screens, glycerol stocks were thawed and glycerol was removed by pelleting cells and washing with fresh SCD-Ura three successive times. Cells were transferred into a custom turbidostat and grown at constant turbidity (target OD_600_ = 1.0) in the appropriate media [19]. For the plasmid-based CiBER-seq screen comparing P(Z3) to P(Z4) and the CL1 CiBER-seq screen with Z3PM-CL1 and Z4PM, cells were grown in SCD-Ura supplemented with 200 nM or 135 nM progesterone, respectively. For the NMD reporter screen, cells were grown in SCD-Ura with no hormone. Cells were grown for 16 + h and 10 OD_600_ units were collected by centrifugation as a pre-induction sample. Anhydrotetracycline (Takara Bio, 631310) was then added to the growth chambers and the media reservoir to a final concentration of 250 ng/mL, and cells were grown for ~ 9 h before collecting 10 OD_600_ units for a post-induction sample. For the NMD CiBER-seq screen, cycloheximide (Sigma-Aldrich, C1988) was added to the media to a final concentration of 100 µg/mL and cells were grown for another hour before collecting 10 OD_600_ units. After removing the media, all samples were flash frozen in LN_2_ and stored at − 80 °C until ready for library preparation. All CiBER-seq screens were performed in two biological replicates from independent library transformations.

### Barcode isolation, library preparation, and deep sequencing

RNA barcodes were isolated from cell pellets by phenol–chloroform extraction and ethanol precipitation followed by cDNA synthesis and library construction with low cycle PCR. Cell pellets were resuspended in 440 µL of 50 mM NaOAc pH 5.2, 10 mM EDTA, 1% SDS (v/v) and 400 µL phenol–chloroform and heated with agitation at 65 °C for 15 min before chilling on ice for 5 min. The sample was centrifugated at 20,000 g at 4 °C for 5 min, washed twice with 400 µL chloroform, and the aqueous phase was transferred to a new RNAse-free tube. RNA was precipitated by adding 50 µL of 3 M NaOAc pH 5.2, 1 µL GlycolBlue (Invitrogen, AM9515), and EtOH to 80% (v/v). The RNA was pelleted by centrifugation at 20,000 g at 4 °C for 10 min, washed once with 500 µL ice-cold 70% EtOH, and air dried for 5 min before resuspending in 50 µL water and quantified by nanodrop. 20 µg of RNA was treated with TURBO DNAse (Invitrogen, AM2238) in 100 µL volume for 30 min at 37 °C and then further purified by spin column (Zymo Research, R1013) according to the manufacturer’s instructions. After DNAse treatment of the cycloheximide treated samples, 10 µg of RNA was treated with 1 µL Xrn1 (NEB, M0338S) for 1 h at 37 °C to remove decapped, 5′ phosphorylated mRNAs before being purified by spin column. Following RNA isolation, 4 µg of each sample was reverse transcribed using dT priming with Protoscript II (NEB, M0368L) and subsequently treated with 0.5 µL each of Rnase H and Rnase A (Thermo Fisher, EN0531 and NEB, M0297S) for 30 min at 37 °C. cDNA was purified by spin column clean up (Qiagen, 28104) and used as input for step-1 PCR with oJL923, oJL924, and oJL1058 along with oJL555 for YFP, CFP, and mScarlet amplicons, respectively. All PCR was performed in 50 µL reactions with a 95 °C initial denaturation for 3 min, followed by 7–10 cycles of 98 °C for 20 s, 60 °C for 15 s, and 72 °C for 10 s, with a final extension of 72 °C for 2 min using Q5 Hot Start High-Fidelity DNA Polymerase (NEB, M0493L). We used 7 cycles for all amplicons, except those from the CL1-YFP sample, which were expressed at low levels and required 10 cycles. Following step-1 PCR, samples were purified by spin column and eluted in 50 µL water. Half the elution was used as input for step-2 PCR with Illumina-compatible dual index primers containing unique dual indexes (provided by UCSF CAT), using 8 cycles for the CL1-YFP sample and 7 cycles for all other transcripts. Amplicons were then purified using AMPure XP beads (Beckman Coulter, A63882) using a 1:1 bead:PCR ratio, washed once with 180 µL 80% EtOH and eluted in 15 µL water. Samples were quantified by qPCR using DyNAmo HS SYBR Green qPCR (ThermoFisher, F410L) on a Stratagene Mx300P instrument, pooled, and further analyzed by automated electrophoresis using an Agilent Tapestation 2200. Pools were sequenced on an Illumina Novaseq-X with single-end 100 bp or paired-end 150 bp reads.

For extraction of DNA barcodes, pellets were thawed and plasmids were isolated using the Zymoprep Yeast Plasmid Miniprep II kit (Zymo Research, D2004) with minor modifications: The pellet was resuspend in 1 mL of solution-1 and 30 µL zymolase was added, digesting for 3 h with agitation at 37 °C. The reaction was then split into 5 tubes and then prepared separately according to standard protocol. After pelleting precipitant, the samples were loaded successively onto a single spin column to minimize nonspecific plasmid loss and then washed and eluted according to standard protocol. YFP barcodes were first amplified for 8 cycles using oPD573 and oJL521 and then purified by spin column. Half the elution was used as input to another PCR to add overhangs for sequencing primers using oJL923 and oJL555 for 7 cycles. The sample was again purified by spin column and sequencing primers were added as described above using another 7 cycles of amplification. Samples were then purified, quantified, and analyzed as described above.

### Analysis of barcode counts and determining guide effects

Illumina sequencing reads were trimmed with cutadapt, mapped to guides using bowtie2 with the appropriate barcode sequences, and counts were extracted from alignments with samtools [58–60]. Barcodes with fewer than 5 reads in the pre-induction sample were discarded and excluded from downstream analysis. All raw barcode counts are available at the NCBI Gene Expression Omnibus (GSE268777). Reads for each condition were analyzed with an establish general linear model framework using the *mpra* package in R, summing counts for each guide across the independent barcodes [20]. Specifically, linear models were implemented to measure the change in barcode ratio between the pre- and post-guide induction samples while accounting for variance between the two biological replicates. Guides with a false discovery rate corrected *p*-value < 0.01 were scored as statistically significant, and adjusted *p*-values < 10^−50^ were set to 10^−50^ for display. All screens assessed the change in barcode counts after guide expression, using the pre-anhydrotetracycline sample as the baseline. For the cycloheximide comparison, the post-anhydrotetracycline sample was used as the baseline.

### Gene ontology analysis

Gene ontology analysis was performed using PantherDB to calculate statistical overrepresentation using the Fisher’s exact test and significance was assessed with a Bonferroni corrected *p*-value < 0.05 [61]. Full GO terms with fold enrichment and calculated significance are displayed. We did not display comparisons that have no significant GO terms.

### Bxb1-mediated integration

Two independent clonal isolates of yeast containing the integrated Bxb1 landing pad derived from pNTI829 were each transformed with 100 fmol ARS/CEN or Bxb1 donor plasmid DNA as described above. The ARS/CEN transformation used 50 fmol (175 ng) each pNTI854 and pNTI855, while the Bxb1 donor transformation used 50 fmol (140 ng) each pNTI832 and pNTI833. A small fraction of yeast from each transformation were taken for serial dilution and plating on selective SCD-Ura media to estimate transformation efficiency. The rest of the transformation was inoculated into 50 mL pre-warmed SCD-Ura at an OD_600_ of ~ 0.1 and incubated for 22 h at 30 °C with shaking and aeration, at which point the OD_600_ had reached 1.9 for the ARS/CEN transformation and 0.28 for the Bxb1 transformation. Yeast were then diluted into 20 mL of fresh pre-warmed SCD-Ura to achieve an estimated OD_600_ of 0.025 and growth was continued at 30 °C with shaking and aeration for another 17 h. A sample of yeast was fixed for flow cytometry, as above. Non-selective cultures were established by diluting yeast into pre-warmed YEPD at an OD_600_ of ~ 0.1 and growing cells for an additional 6 h with shaking and aeration, at which point they reached an OD_600_ of ~ 1.5. An additional sample of yeast were fixed at this point and measured on a BD LSR Fortessa X20 Analyzer. yECitrine and yEmScarlet-I fluorescence was measured for 100,000 total events per sample, and gating and analysis was performed using *flowCore* in R [54].

### RT-qPCR

Effects of individual guides were validated by performing RT-qPCR on 2–3 biological replicates of the appropriate yeast strain (Additional file 2: Table S1). Yeast were grown overnight to saturation in SCD-Ura and then back diluted to OD_600_ = 0.1 into SCD-Ura in a 96 well deep-well block and covered with a Breathe-Easy gas permeable seal (BEM-1, Diversified Biotech), adding 135 nM progesterone if needed. When cells reached OD_600_ = 0.5, anhydrotetracycline was added to a final concentration of 250 ng/mL and cells were grown for 16 h. The following morning, yeast were further back diluted to OD_600_ = 0.5 and grown for 6 more hours in SCD-Ura + 250 ng/mL anhydrotetracycline, and progesterone when appropriate. For validation of individual guides, yeast were collected by centrifugation and RNA was isolated by the formamide-EDTA approach [62]. Briefly, yeast pellets were resuspended in 40 µL FAE solution (98% formamide, 10 mM EDTA), gently mixed, and heated at 70 °C for 10 min. Samples were then quickly vortexed, pelleted by centrifugation at 16,000 g for 2 min at room temperature, and 35 µL of the supernatant was transferred to a new tube. RNA was further purified by spin column (Zymo Research, R1018) and DNA was removed by on-column DNAse digestion according to the manufacturer’s protocol. RNA was then quantified by nanodrop and ~ 500 ng of RNA was used as input for cDNA synthesis using dT priming with Protoscript II Reverse transcriptase (NEB, M0368S). cDNA was then further diluted twofold in water and used as input for qPCR as described above. All qPCR was performed with oJL555 with oJL923, oJL924, and oJL1058 to detect YFP, CFP, and mScarlet, respectively, while *DCP1* expression was assessed with oJL1433 and oJL1434. Quantification of the NMD reporter in the pre, post, and cycloheximide samples was performed on cDNA isolated from the CiBER-seq screens for the YFP and mScarlet transcripts.

### Growth curves

Yeast expressing the appropriate transcription factor and promoter combination were grown overnight in SCD-Ura and backdiluted to OD_600_ = 0.1 the following morning. Cells were grown to OD_600_ = 0.5 and then further backdiluted to OD_600_ = 0.05 in SCD-Ura with 200 nM progesterone or a matched volume of ethanol as a control. 100 µL of cells were transferred to a 96 well U-bottom plate (Corning, 3788), sealed with a Breathe-Easy gas permeable seal (Diversified Biotech, BEM-1), and grown at 30 °C with constant agitation in a Tecan Spark plate reader while measuring the OD_600_ every 5 min.

## Supplementary Information

Additional file 1: Supplementary figures.

Additional file 2: Table S1. Yeast strains, plasmids, and primers used in this study.

## Data Availability

CiBER-seq data were deposited with the NCBI Gene Expression Omnibus with under accession number GSE268777 [63] and are available at the following URL: https://www.ncbi.nlm.nih.gov/geo/query/acc.cgi?acc=GSE268777. Analysis software is available on GitHub [64] at the following URL under an MIT license: https://github.com/ingolia-lab/CiBERopt_main. and the version used in the manuscript is available from Zenodo [65] at the following URL:10.5281/zenodo.15098700. CiBER-seq data from previous studies were deposited with the NCBI Sequence Read Archive under accession number PRJNA578818 [66] and are available at the following URL: https://www.ncbi.nlm.nih.gov/bioproject/?term=PRJNA578818.
