# Supplementary material for: Precise measurement of molecular phenotypes with barcode-based CRISPRi systems
Source: Genome Biol. 2025 May 25;26:142. doi: 10.1186/s13059-025-03610-w (PMC12103760; doi:10.1186/s13059-025-03610-w)
Supplement: Supplementary file 1 — Additional file 1: Supplementary figures. [file 13059_2025_3610_MOESM1_ESM.pdf]

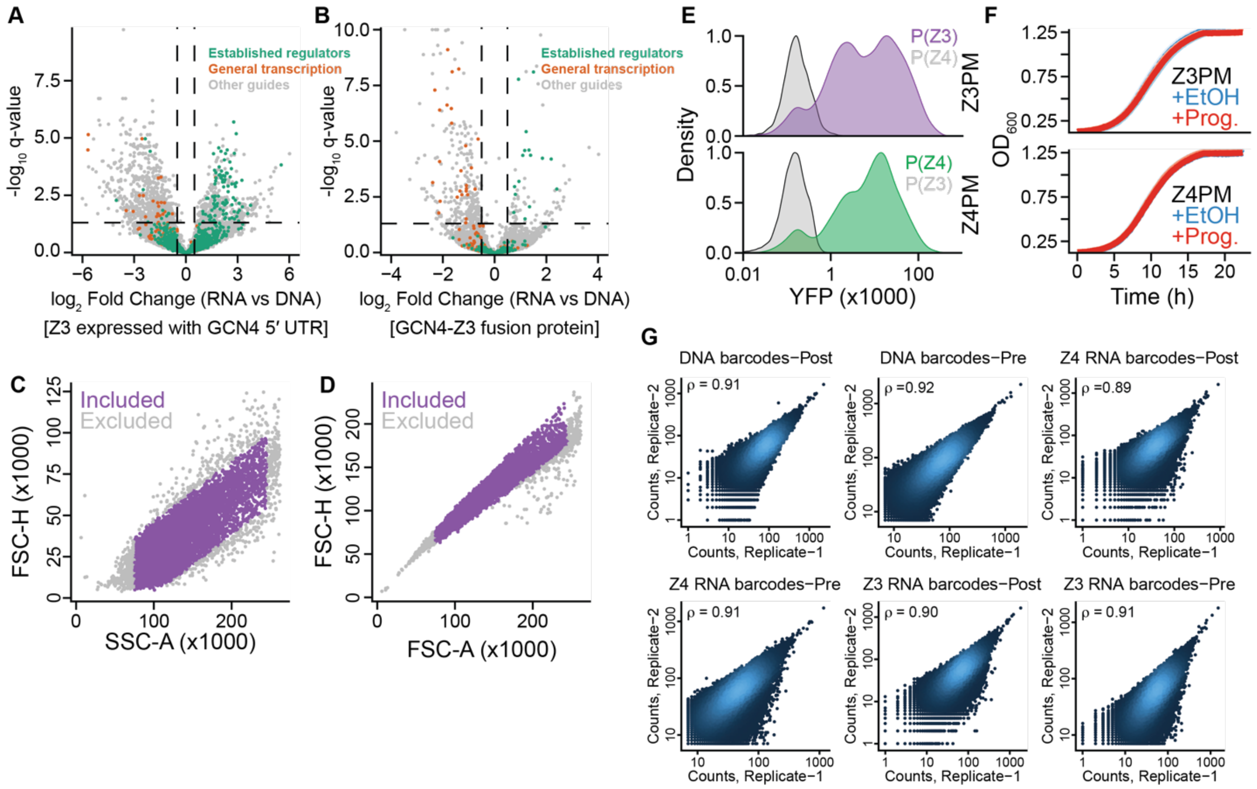

Fig. S1: Evaluation of both previous CiBER-seq screens and Z3PM and Z4PM-driven reporter expression. (A-B) Analysis of previously published genome-wide CiBER-seq screens for regulators of the integrated stress response(5). Z3 transcription factor expressed with the GCN4 5′ UTR **(A)** or fused to the GCN4 coding sequence **(B)**. Each point is a single guide and colored based on molecular function in panel legend. Guide activity was measured using RNA-to-DNA barcode ratios. Significant and robust guides were assessed by a q-value < 0.05 and > 0.5 $\log_2$ fold change, which is represented by dashed lines. **(C-D)** Representative gating criteria for forward and side scatter in all flow cytometry analysis. **(E)** Representative raw flow cytometry histograms of Z3PM or Z4PM driving YFP expression from P(Z3) or P(Z4) at 200 nM progesterone. **(F)** Raw growth curves from yeast containing Z3PM or Z4PM and expressing YFP from the cognate promoter, with 200 nM progesterone or matched volume of ethanol in SCD-Ura (n=3). **(G)** Correlation between barcode counts for each replicate, with Pearson's correlation displayed in panel.

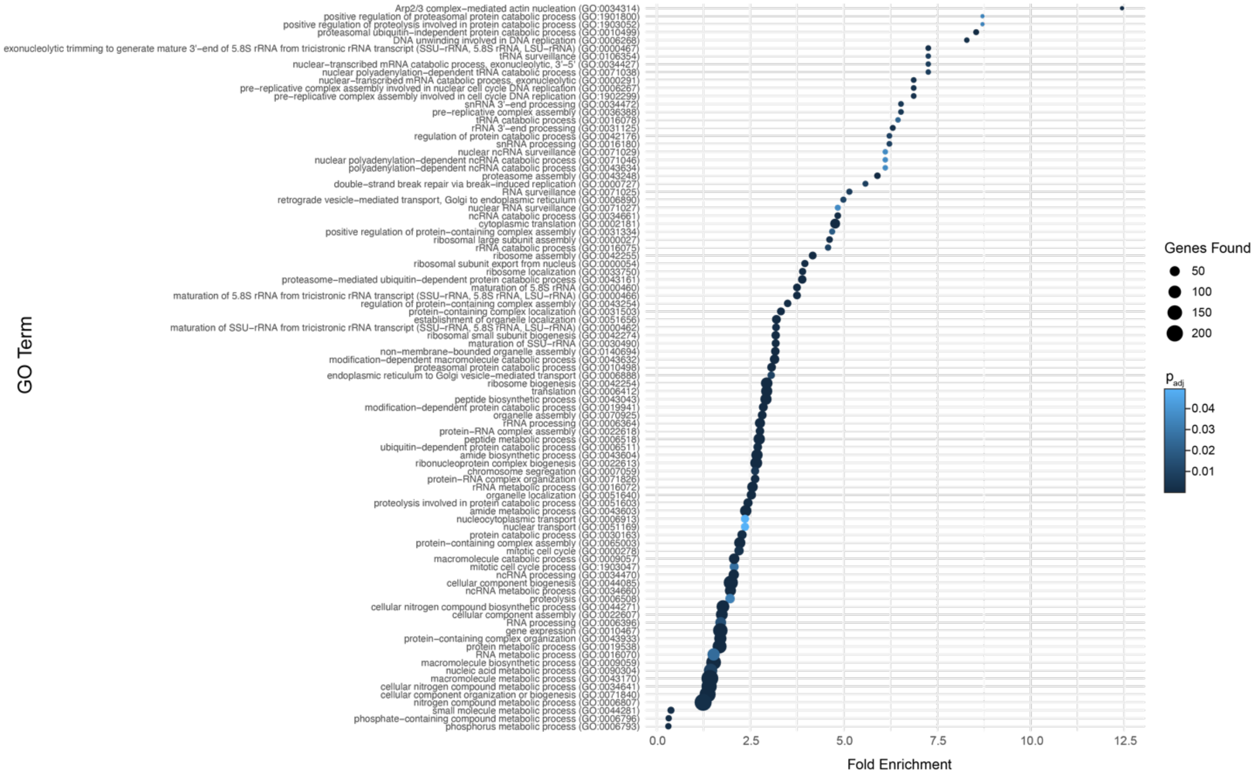

Fig. S2: Analysis of significant processes in DNA-normalized CiBER-seq. Gene ontology terms for guides that significantly increased barcode expression in Fig. 1G, with significance (q < 0.05) calculated from the Fisher's exact test with the Bonferroni correction. There were no significant gene ontology terms for guides that decreased barcode expression in the RNA-to-DNA comparison.

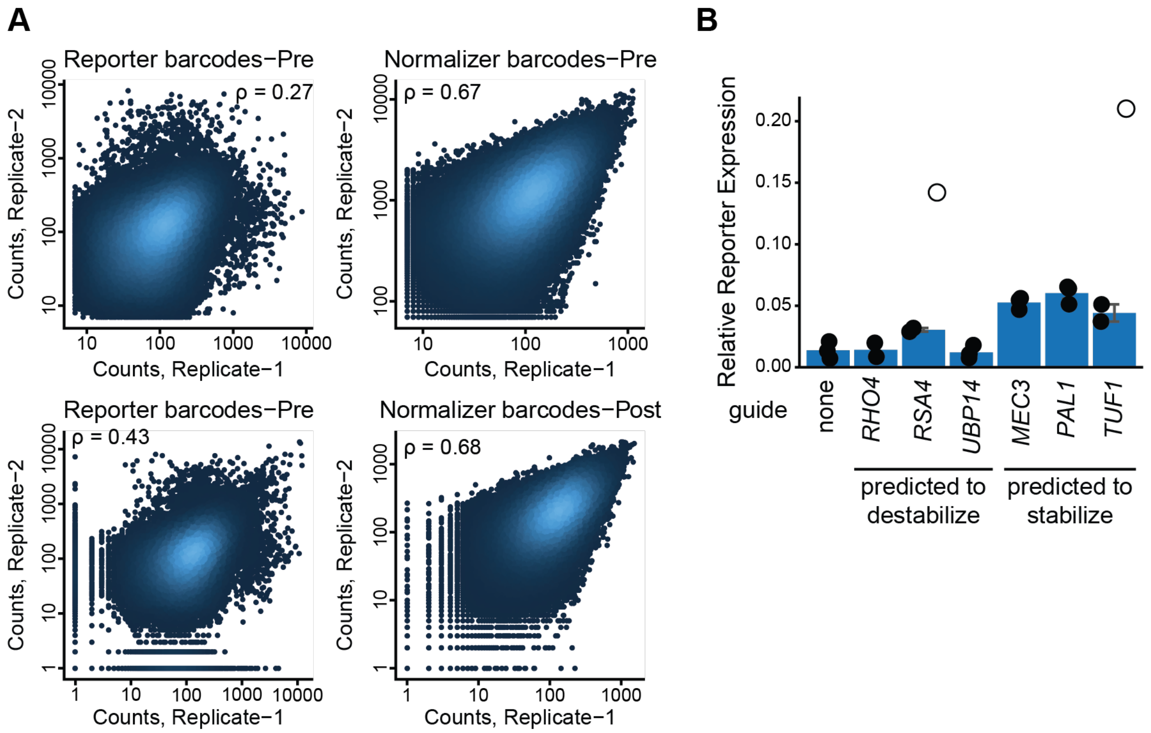

Fig. S3: CiBER-seq analysis of regulators of CL1 degron turnover. (A) Correlation between barcode counts for each replicate, with Pearson's correlation displayed in panel. **(B)** RT-qPCR of reporter transcript driven by Z3PM-CL1 with guides induced compared to normalizer transcript (n=2-3). CiBER-seq nominated *RHO4*, *RSA4*, and *UBP14* as genes whose knockdown further destabilized the reporter and are likely false positives. Effect of the guide inferred by CiBER-seq is labelled below. We confirmed that induction of the other guides increased reporter expression. Mean calculated from biological replicates displayed as black points. Outliers excluded from analysis shown as white points.

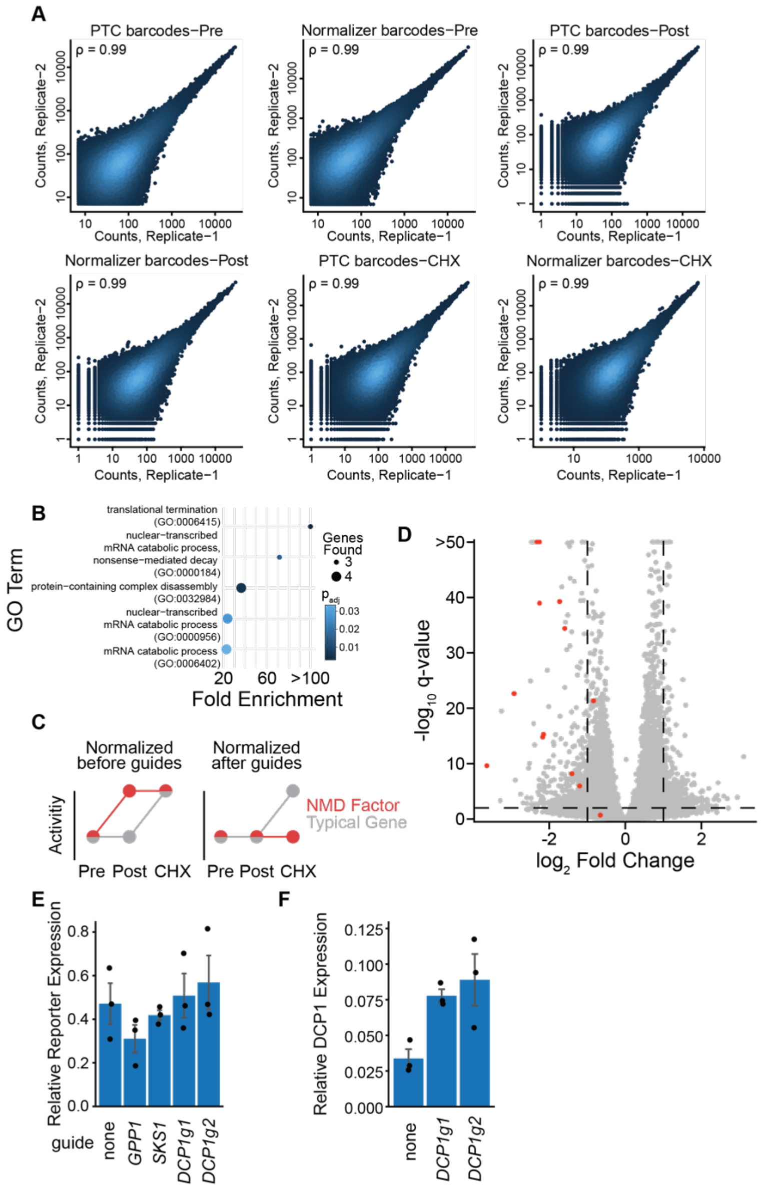

Fig. S4: CiBER-seq profiles the requirement of activate translation for NMD activity. (A) Correlation between barcode counts for each replicate, with Pearson's correlation displayed in panel. **(B)** Gene ontology enrichment analysis of guides that stabilize the PTC containing reporter compared to all guides from Fig. 4E. Significance (q < 0.05) was assessed with Fisher's exact test with the Bonferroni correction. No significant gene ontology terms were found for guides that appear to further destabilize the reporter. **(C)** Schematic of expected guide activities based on the comparisons in linear models. NMD factors should have lower activity than a typical gene in the cycloheximide treatment, because the reporter harboring the PTC is not further stabilized by drug treatment. **(D)** Analysis of genome-wide CiBER-seq screen for NMD factors after cycloheximide treatment, normalized to post-guide induction. Guides that significantly stabilized the NMD reporter in Fig. 4E are labeled in red. Dashed lines represent q-values < 0.01 and > 1 $\log_2$ fold change. **(E)** RT-qPCR of PTC-containing mRNA compared to normalizer with guides induced (n=3). CiBER-seq nominated *GPP1* and *SKS1* as potential genes whose knocked enhanced NMD. Two different guides targeting *DCP1* are displayed **(F)** RT-qPCR of DCP1 mRNA expression with two different guides targeting *DCP1* compared to a no-guide control.
